# Supplementary material for: Building safer and more resilient cities in China: A novel approach using a dynamic nonhomogeneous Gray model for data-driven decision-making
Source: PLoS One. 2024 Dec 5;19(12):e0310554. doi: 10.1371/journal.pone.0310554 (PMC11620441; doi:10.1371/journal.pone.0310554)
Supplement: Attachment — Submitted filename: PONE-D-24-16340_Review.pdf [file pone.0310554.s001.pdf]

Title: Building Safer and More Resilient Cities in China: A Novel Approach Using a Dynamic Nonhomogeneous Gray Model for Data-Driven Decision-Making

This study provides an interesting topic with a concise overview of the research on building safety within the context of urban resilience in China. I think the main point of this research wants to convey the importance of urban resilience in the face of rapid urbanization and the necessity of developing cities that can foresee risks, reduce disaster impacts and recover swiftly from crises. The proposed dynamic nonhomogeneous gray model (DNMGM(1,1)) is clearly presented as the core methodology. Overall, the manuscript is well written, and the implementation and the experiment are an interesting contribution, but there is little original method, and research problem and significance are ambiguous. I have the following detailed concerns:

Abstract: The abstract presents a compelling case for the use of the DNMGM (1,1) model in enhancing urban resilience through better prediction and planning in building safety. While the abstract is largely comprehensive, it could briefly mention any limitations of the study or areas for future research. How the findings can be implemented by urban planners or policymakers might further enhance the practical relevance of the study?

1. **Introduction:**

This introduction effectively highlights the importance of the construction industry, the significant economic contribution of this sector and the pressing concerns related to construction safety. It provides a well-rounded context by discussing the economic impact, employment generation, and the societal challenges associated with construction safety incidents.

Areas for improvement: The introduction is dense with information and could benefit from clearer, more concise language in some parts. Simplifying the explanation of complex models and their limitations could enhance readability. While the introduction references several studies, integrating these references more seamlessly into the narrative could improve the flow. For instance, directly linking specific studies to the claims made about model performance can strengthen the argument. Explicitly stating the research gap (i.e., the need for a more accurate model for predicting construction fatalities) and the objective of the paper (introducing the DNMGM(1,1) model) earlier in the introduction could provide clearer direction. Briefly mentioning how the new model could be practically implemented in the construction industry and its potential real-world impact would add depth and relevance. Some transitions between paragraphs could be smoother. For example, moving from the general importance of construction safety to specific predictive models feels abrupt. A clearer structure with subheadings might help in organizing the content more logically. By enhancing clarity, conciseness, and integrating practical implications, the introduction can be further strengthened.

2. **Approximate inhomogeneous dynamic GM(1,1) modeling process:**

While this section is informative, some parts are dense and could be more concise. Simplifying the language and breaking down complex ideas into shorter sentences could improve readability. The transitions between discussing static models, dynamic models, and the specifics of the DNMGM(1,1) model could be smoother. Clearer subheadings might help guide the reader through the different sections. The mention of Fig. 1 is helpful, but a brief description or summary of what the figure illustrates would aid in understanding.

The theoretical justification for the DNMGM(1,1) model is strong, a brief discussion on how this model could be practically implemented in the construction industry would add value. For example, mentioning specific steps or tools for integrating this model into existing safety protocols could enhance its practical relevance. Providing hypothetical or actual examples of how the DNMGM(1,1) model has improved prediction accuracy in comparison to other models would make the argument more compelling.

3. **Forecasting of the death risk in China:**

This section provides a good overview of the historical trends in construction accident fatalities in China and the rationale for using the gray prediction model. Redundant phrases should be eliminated. Here, authors briefly mentioned various indicators but does not explain why fatalities are the primary focus. A brief justification would be helpful. More detailed description of what Fig. 2 shows would help readers who cannot see the figure immediately. Moreover, providing more context about the data source and type would strengthen the credibility of the data.

4. **Static prediction results and analysis:**

Some sentences are lengthy and complex, which can affect readability. The explanation of what Fig. 3 and Fig. 4 depict could be more detailed. This would help readers understand the visual data better. Providing a bit more context about why certain models performed better or worse could enhance the reader's understanding of the underlying reasons for these differences. The transition between discussing the performance of the models and the final conclusion could be smoother.

5. **Dynamic prediction results and analysis:**

Ensure that all technical terms and abbreviations, such as MAPE and DNGM(1,1), are clearly defined when first introduced. Include direct references to the figures in the text to guide the reader (e.g., "As shown in Fig. 5"). Provide a brief explanation of why MAPE is used as the evaluation metric and how it is calculated. Expand the description of each model (GM, DNGM, MGM, DNMGM) to provide more context on their differences and similarities.

By incorporating these revisions, the section will be more informative and easier for readers to understand the significance and implications of the dynamic adaptive capability and generalizability of the DNMGM(1,1) model.

Ensure consistent use of model names (for e.g. Gray/ Grey) and terms throughout the text. Include direct references to figures and tables within the text to guide the reader (e.g., "As shown in Table 1"). Overall, improve the structure and flow of the sections to enhance readability and logical coherence, ensuring smooth transitions between paragraphs and sections. Correct minor typographical errors (e.g., "is or observed data" should be "or observed data is" and Instead of he "The").
