# Supplementary material for: Building safer and more resilient cities in China: A novel approach using a dynamic nonhomogeneous Gray model for data-driven decision-making
Source: PLoS One. 2024 Dec 5;19(12):e0310554. doi: 10.1371/journal.pone.0310554 (PMC11620441; doi:10.1371/journal.pone.0310554)
Supplement: Attachment — Submitted filename: Response to Reviewers.docx [file pone.0310554.s002.docx]

**Dear Editor,**

Thank you for providing the reviews for our paper entitled " Building Safer and More Resilient Cities in China: A Novel Approach Using a Dynamic Nonhomogeneous Gray Model for Data-Driven Decision-Making."

We deeply appreciate the time and attention the reviewers have devoted to our manuscript. Their valuable comments have significantly contributed to the refinement of our paper and provided essential guidance that has shaped our research. We have thoroughly revised our manuscript based on their suggestions and your specific editorial advice. We have carefully considered each comment and made detailed corrections, *which are highlighted* in blue in the revised manuscript. Specifically, we have made the following changes:

**Page 2**, lines 18-21; **Page 5**, lines 14-25; **Page 6**, lines 12-13; **Page 7**, lines 21-28;

**Page 8,** lines 1-3; **Page 10**, lines 10-12; **Page 12**, lines 10-13; **Page 12**, lines 15-18;

**Page 12**, lines 23-27; **Page 13**, lines 1-21; **Page 14**, lines 10-18; **Page 15**, lines 5-14;

**Page 15**, lines 18-27; **Page 17**, lines 3-11; **Page 17**, lines 15-28; **Page 18**, lines 1-12;

**Page 19**, lines 13-23; **Page 25**, lines 8-17; **Page 26**, lines 3-15; **Page 27**, lines 6-28;

**Page 28**, lines 1-16; **Page 30**, lines 4-11;

We hope these revisions meet your requirements and that our manuscript fully resonates with the journal's focus on building safety. Thank you once again for the opportunity to enhance our manuscript. We are grateful for the editorial guidance and hope our revised submission will be favorable. Please do not hesitate to contact us should you need further modifications or have additional feedback.

Best Regards,

Sincerely,

Rui Feng.

**The following are responses to each point raised by the academic editor and reviewer(s).**

**Reviewer #1:**

*This study provides an interesting topic with a concise overview of the research on building safety within the context of urban resilience in China. I think the main point of this research wants to convey the importance of urban resilience in the face of rapid urbanization and the necessity of developing cities that can foresee risks, reduce disaster impacts and recover swiftly from crises. The proposed dynamic nonhomogeneous gray model (DNMGM(1,1)) is clearly presented as the core methodology. Overall, the manuscript is well written, and the implementation and the experiment are an interesting contribution, but there is little original method, and research problem and significance are ambiguous. I have the following detailed concerns:*

**Comment 1:**

*Abstract: The abstract presents a compelling case for the use of the DNMGM (1,1) model in enhancing urban resilience through better prediction and planning in building safety. While the abstract is largely comprehensive, it could briefly mention any limitations of the study or areas for future research. How the findings can be implemented by urban planners or policymakers might further enhance the practical relevance of the study?*

**Response 1：**

Thank you for your thoughtful review of our manuscript. We appreciate your constructive feedback and have made revisions to the abstract accordingly to address your points. In response to your suggestion, we have added a brief mention of the study's limitations and future research directions, as well as how the findings can be practically implemented by urban planners and policymakers. Specifically, we now add at the end:

Page 2, lines 18-21:

Specific interventions and policy frameworks based on this study by urban planners and policymakers can promote resilient urban development. Future efforts should strive to enhance its robustness and adaptability in different fields.

**Comment 2:**

*Introduction:*

*This introduction effectively highlights the importance of the construction industry, the significant economic contribution of this sector and the pressing concerns related to construction safety. It provides a well-rounded context by discussing the economic impact, employment generation, and the societal challenges associated with construction safety incidents.*

**Comment 2-(1):** *Areas for improvement: The introduction is dense with information and could benefit from clearer, more concise language in some parts. Simplifying the explanation of complex models and their limitations could enhance readability. While the introduction references several studies, integrating these references more seamlessly into the narrative could improve the flow. For instance, directly linking specific studies to the claims made about model performance can strengthen the argument.*

**Comment 2-(2):** *Explicitly stating the research gap (i.e., the need for a more accurate model for predicting construction fatalities) and the objective of the paper (introducing the DNMGM(1,1) model) earlier in the introduction could provide clearer direction. Briefly mentioning how the new model could be practically implemented in the construction industry and its potential real-world impact would add depth and relevance.*

**Comment 2-(3):** *Some transitions between paragraphs could be smoother. For example, moving from the general importance of construction safety to specific predictive models feels abrupt. A clearer structure with subheadings might help in organizing the content more logically. By enhancing clarity, conciseness, and integrating practical implications, the introduction can be further strengthened.*

**Response 2：**

Thank you for your insightful comments and feedback on our manuscript. We have directly addressed each of your comments and suggestions, incorporating them into the revised manuscript where appropriate. We have carefully considered your suggestions and have revised the manuscript accordingly. We believe that the revisions have significantly improved the logical flow and clarity of our presentation.

Specifically, we have:

**Response 2-(1):** Simplified explanation of complex models and limitations: We have organized the chapters to follow a more logical progression and simplified the language. This helps to clarify the overall narrative and makes the argument more convincing. Example:

Page 7, lines 21-24:

Nevertheless, despite their advantages, the linear characteristics of the classical GM(1,1), DGM(1,1), and even the improved DNGM(1,1), IDGM(1,1), and DDGM(1,1) models may still lead to inaccurate predictions in specific practical contexts.

**Response 2-(2):** Thank you for your useful advice. In response to your suggestion, we have clarified the need for a more accurate model for predicting building mortality immediately after giving the background. It also briefly mentioned how the practical implementation of the new model in the construction industry and its potential real-world impact would add depth and relevance.

Page 5, lines 16-25:

The key to truly enhancing the resilience of resilient cities to withstand emergencies lies in establishing and implementing an efficient accident prediction and prevention mechanism and introducing a more accurate fatality prediction model that predicts the durability of buildings under extreme conditions. The practical implementation of this model to optimize safety through data-driven decision-making has the potential real-world impact of significantly improving the safety and security of residents and guiding the transformation and upgrading of the construction industry to promote safer and more sustainable urban development.

**Response 2-(3):** Strengthened logical connections: We have added transitional phrases and sentences where necessary to strengthen the logical connections between paragraphs and sections. This has improved the flow of ideas and made the manuscript easier to follow. For example:

Page 6, lines 12-13:

However, construction safety events, characterized by complexity and uncertainty, often manifest as approximately non-homogeneous sequences.

**Comment 3:**

*Approximate inhomogeneous dynamic GM(1,1) modeling process:*

**Comment 3-(1):** *While this section is informative, some parts are dense and could be more concise. Simplifying the language and breaking down complex ideas into shorter sentences could improve readability. The transitions between discussing static models, dynamic models, and the specifics of the DNMGM(1,1) model could be smoother.*

**Comment 3-(2):** *Clearer subheadings might help guide the reader through the different sections.*

**Comment 3-(3):** *The mention of Fig. 1 is helpful, but a brief description or summary of what the figure illustrates would aid in understanding.*

**Comment 3-(4):** *The theoretical justification for the DNMGM(1,1) model is strong, a brief discussion on how this model could be practically implemented in the construction industry would add value. For example, mentioning specific steps or tools for integrating this model into existing safety protocols could enhance its practical relevance. Providing hypothetical or actual examples of how the DNMGM(1,1) model has improved prediction accuracy in comparison to other models would make the argument more compelling.*

**Response 3：**

Thanks for your very pertinent advice. I have made the following modifications in response to your suggestion:

**Response 3-(1):** Simplify the language. Examples are as follows:

①The original sentence: The factors influencing the forecasting results vary with the changes in the system state. Therefore, when new modeling data are continuous in time, the original data do not need to be used in entirety. Only need to extract new data and replace the oldest data according to a certain data size.

After simplification (Page 10, lines 10-12):

Factors affecting forecasts change with system state. When new modeling data is continuous, the original data is unnecessary. Extract new data and replace the oldest based on a specified size.

②The original sentence: DNMGM(1,1) combines the characteristics of both DNGM(1,1) and MGM(1,1). It introduces parameter estimation in the whitening differential equations and applies the metabolic concept. This model is the most complex among the four models, yet it is also the most powerful, capable of handling more complex data patterns.

After simplification (Page 12, lines 10-13):

DNMGM(1,1) merges DNGM(1,1) and MGM(1,1) traits. It estimates parameters in whitening differential equations and uses metabolism. The most complex model is also the most capable of handling intricate data patterns.

③The original sentence: The classic GM model only uses a random dataset size, and the accuracy improvement achieved by using the optimal dataset size is not considered. By determining the optimal dataset size for the simulated data, the internal data trends can be identified; that is, different data quantities may yield different prediction results, and the prediction efficiency can vary.

After simplification (Page 12, lines 15-18):

The classic GM model uses a random dataset size, disregarding optimal size for accuracy. Identifying the optimal size for simulated data reveals data trends. Different data amounts yield varied predictions and efficiency.

**Response 3-(2):** Based on your suggestions, I added some transitional statements and divided this section into four subheadings:

Traditional modeling process and its errors、The necessity and introduction process of dynamic modeling、Comparison of characteristics of four models、Application of DNMGM(1,1) model.

**Response 3-(3):** A brief summary of Figure 1:

Page 10-11, lines 18-20 & 1:

Fig. 1 shows a complete process from data preprocessing to model building, parameter estimation, optimal data size determination, and dynamic modeling to improve the accuracy and robustness of time series predictions.

**Response 3-(4):** Thank you for your valuable comments and suggestions on our paper. Based on your feedback, we have made targeted modifications and supplements to the relevant paragraphs further to enhance the practicality and persuasiveness of the paper.

First, we explore in depth the practical implementation value of the DNMGM(1,1) model in the construction industry. We envision concrete steps or tools to integrate this model into existing building safety protocols, aiming to provide scientific guidance to governments to help them adjust safety inputs and optimize the allocation of safety personnel through real-time analysis and prediction of construction accident risk. Such applications can improve the overall safety of the construction industry and significantly reduce accident rates and fatalities.

Secondly, in order to more fully demonstrate the advantages of the DNMGM(1,1) model, we conducted a hypothetical or practical comparative analysis. We compare the prediction accuracy of the DNMGM(1,1) model with other prediction models (such as traditional time series analysis models, machine learning models, etc.). Through comparison, it is found that the DNMGM(1,1) model, with its unique dynamic method and iterative calculation process, captures the nonlinear and unstable characteristics of the data more accurately, thus achieving a significant improvement in the prediction accuracy. These comparative results not only enhance the theoretical value of this study, but also provide strong support for its promotion in practical applications.

We believe that these changes and additions will help improve the paper's usefulness and persuasiveness and make it more in line with the reviewer's expectations and requirements.

The supplementary content is as follows:

Page 12-13, lines 23-27 & 1-22:

In the construction industry, the practical implementation of the DNMGM(1,1) model is of great value. For example, we can imagine integrating this model into existing building safety protocols, through specific implementation steps or developing specialized tools, to analyze and predict the risk of building accidents in real-time, thereby providing scientific guidance to governments to help them adjust safety investments and optimize the allocation of safety personnel. Such applications can improve the overall safety of the construction industry and significantly reduce accident rates and fatalities.

In order to further demonstrate the advantages of the DNMGM(1,1) model, we conducted a hypothetical or practical comparative analysis. Compared with other prediction models (such as traditional time series analysis models, machine learning models, etc.), the DNMGM(1,1) model captures the nonlinearity and instability characteristics of the data more accurately through its unique dynamic method and iterative calculation process, thus achieving a significant improvement in prediction accuracy. These comparative results not only enhance the theoretical value of this study, but also provide strong support for its promotion in practical applications.

**Comment 4:**

*Forecasting of the death risk in China:*

**Comment 4-(1):** *This section provides a good overview of the historical trends in construction accident fatalities in China and the rationale for using the gray prediction model. Redundant phrases should be eliminated.*

**Comment 4-(2):** *Here, authors briefly mentioned various indicators but does not explain why fatalities are the primary focus. A brief justification would be helpful.*

**Comment 4-(3):** *More detailed description of what Fig. 2 shows would help readers who cannot see the figure immediately.*

**Comment 4-(4):** *Moreover, providing more context about the data source and type would strengthen the credibility of the data.*

**Response 4：**

Thank you very much for your advice. I have added to your suggestion:

**Response 4-(1) :** We checked statements and removed redundant vocabulary to make the article more readable.

**Response 4-(2) :** Explain that death is the main focus in the indicators:

Page 14, lines 11-18:

The death toll directly reflects the severity of the accident and the direct impact on human life, which is one of the most direct and significant consequences of the accident. It is not only an important factor in classifying the accident level, but it also helps to classify accident management so that the relevant departments can quickly take corresponding emergency measures and follow-up treatment according to the accident level. At present, safety guidance is still facing challenges, especially in curbing major accidents and reducing a large number of deaths.

**Response 4-(3):** Regarding the detailed description you mentioned in Figure 2, we fully understand your concern to ensure that the paper's content is clear and understandable to readers who cannot immediately see the figure.

After carefully considering your feedback, we recognize that although we have described Figure 2 in the main body, there may indeed be some readers who have difficulty fully understanding the meaning and trends behind the data without looking directly at the image. Therefore, we decided to adjust the position of the image to make it more visible in the text and easy for readers to find and view as they read. This adjustment is intended to enhance the reader's reading experience so that they can more intuitively understand the information shown in Fig. 2.

**Response 4-(4):** Enhance the credibility of data: In order to enhance the credibility of the data, we have explained the source of the data before the data analysis and processing.

Page 15, lines 6-15:

The data used in this paper are from the official statistical yearbook of the Ministry of Housing and Urban-Rural Development of China. The statistical yearbook is regularly compiled and released by authoritative institutions, aiming to comprehensively and systematically reflect the development status, changing trends, and existing problems in the field of housing and urban and rural construction in China. Our data is carefully screened and collated to ensure its accuracy, authority, and timeliness. These data not only provide a solid foundation for the research of this paper, but also further enhance the credibility and persuasiveness of the research conclusions.

**Comment 5:**

*Static prediction results and analysis:*

**Comment 5-(1):** *Some sentences are lengthy and complex, which can affect readability.*

**Comment 5-(2):** *The explanation of what Fig. 3 and Fig. 4 depict could be more detailed. This would help readers understand the visual data better.*

**Comment 5-(3):** *Providing a bit more context about why certain models performed better or worse could enhance the reader's understanding of the underlying reasons for these differences.*

**Comment 5-(4):** *The transition between discussing the performance of the models and the final conclusion could be smoother.*

**Response 5：**

Thank you very much for your advice. In response to your suggestion, we have made the following modifications:

**Response 5-(1) :** We checked statements and removed redundant vocabulary to make the article more readable.

**Response 5-(2):** Additional notes to Figure 3 are provided below. Also, see Response 5-(3) for additions to Figure 4.

Page 15-16, lines 19-27 & 1-2:

The results are shown in Fig. 3, which visually presents the comparison of the data predicted by different models on the number of deaths, while providing a reference base for real data. The solid black line represents the actual number of deaths, and the different colored lines represent the predicted values of the various models. IDGM model (red dashed line) and DDGM model (orange dashed line) are quite different from the reality. It is worth noting that the remaining three dashed lines differ less from reality, but the DNGM model represented by the green dashed line is closest to reality at some extreme points compared to the DGM model (purple dashed line) and the DGM model (blue dashed line), indicating that the DNGM model shows greater accuracy in predicting the actual number of deaths.

**Response 5-(3):** Before explaining the background, an additional description of MAPE was first added:

Page 17, lines 4-7:

In this study, MAPE (Mean Absolute Percentage Error) was selected as the core index to quantify and evaluate the applicability of each model in predicting the number of deaths from construction accidents in China.

Potential reasons for the discrepancy are then explained by adding to Figure 4.

Page 17, lines 10-22:

As shown in Fig. 4, the relative error performance of the five different models in the prediction process are emerged, which provides a basis for in-depth analysis. Upon scrutiny of the forecast results, the GM(1,1) model and the DGM(1,1) model exhibit average relative errors of 15.03% and 15.01%, respectively, in predicting construction-related deaths. The observed average relative error falls within 10-20% range. However, a striking phenomenon is that the relative error values of both models have shown a continuous upward trend since 2009, which is likely to be closely related to non-stationary fluctuations in accident deaths in recent years, especially when the data series lacks strict monotonically increasing characteristics. Traditional exponential growth models such as GM(1,1) and DGM(1,1) are inadequate to capture this complex dynamic change.

**Response 5-(4):** We modify the order of the statements to make them more readable.

Page 17-18, lines 23-28 & 1-8:

In contrast, DNGM(1,1), IDGM(1,1), and DDGM(1,1) models show different performance in prediction accuracy. The DNGM(1,1) model stands out with a MAPE value of 13.73%, which is not only better than the DGM(1,1) model but also its error control is in the ideal range of 10% to 20%, which indicates that the model has advantages in adapting to the complex changing pattern of the death toll of construction safety accidents in China. On the contrary, the MAPE values of IDGM(1,1) and DDGM(1,1) models were 19.79% and 21.59%, respectively, which were significantly higher than those of DNGM(1,1) models, reflecting their deficiency in prediction accuracy. This difference deeply reveals the differences in sensitivity and adaptability of different model structures to specific data features, especially when faced with a data set with a unique downward trend, such as the number of fatalities from construction safety accidents in China.

The results are summarized, and then the meaning is derived. The transition is more natural and logical.

Page 18, lines 9-13:

Further, through comprehensive analysis of the relative error values and their changing trends of the five models, it can be reasonably inferred that under the framework of static models, the DNGM(1,1) model becomes the optimal model for predicting the risk related to building death in China due to its low prediction error and good adaptability. This divergence can be ascribed to the distinctive features of construction safety accidents in China, where the overall trend manifests a decreasing trajectory that gradually stabilizes over time. Consequently, through a comprehensive analysis of the relative error values among the five models, it can be deduced that, within the realm of static models, the DNGM(1,1) model is a more suitable choice for forecasting the risks associated with construction fatalities in China.

**Comment 6:**

*Dynamic prediction results and analysis:*

**Comment 6-(1):** *Ensure that all technical terms and abbreviations, such as MAPE and DNGM(1,1), are clearly defined when first introduced.*

*Include direct references to the figures in the text to guide the reader (e.g., "As shown in Fig. 5").*

*Ensure consistent use of model names (for e.g. Gray/ Grey) and terms throughout the text.*

*Include direct references to figures and tables within the text to guide the reader (e.g., "As shown in Table 1").*

*Correct minor typographical errors (e.g., "is or observed data" should be "or observed data is" and Instead of he "The").*

**Comment 6-(2):** *Provide a brief explanation of why MAPE is used as the evaluation metric and how it is calculated.*

**Comment 6-(3):** *Expand the description of each model (GM, DNGM, MGM, DNMGM) to provide more context on their differences and similarities.*

**Comment 6-(4):** *By incorporating these revisions, the section will be more informative and easier for readers to understand the significance and implications of the dynamic adaptive capability and generalizability of the DNMGM(1,1) model.*

*Overall, improve the structure and flow of the sections to enhance readability and logical coherence, ensuring smooth transitions between paragraphs and sections.*

**Response 6：**

**Response 6-(1):** Thank you for your advice. We have marked or explained technical terms and abbreviations when they first appear. In addition, the "as shown in the figure" and "as shown in the table" are also improved. We have also changed the words gray/grey that appear in the text.

**Response 6-(2):** Note The basis for selecting MAPE as an evaluation indicator:

Page 19, lines 14-20:

MAPE represents prediction errors in percentage terms, allowing us to directly compare prediction performance between different size datasets or different models, as percentage errors eliminate the effect of the magnitude of the data on the evaluation results. At the same time, MAPE is more sensitive to prediction error. Even a small absolute error will result in a high percentage error when the actual value is small.

Description MAPE calculation:

Page 19, lines 20-24:

MAPE is obtained by calculating the percentage difference between the predicted and actual values at each point in time and then taking the average of the absolute values of these percentage errors. This evaluation index directly reflects the accuracy of the forecast.

**Response 6-(3):** 4.2 An extended description of the models and their differences and similarities, and a summary of how they have been improved step by step:

Page 26, lines 6-20: (Summary of the advantages and disadvantages of the previous model)

When comparing previous models with the newly proposed DNMGM model, we find that these models show significant differences and similarities in data processing methods, prediction frameworks, and prediction accuracy. As a basic grey system forecasting tool, GM is famous for its efficient use of small and incomplete information, but limited by its static characteristics, it is difficult to reflect the new data trend in real-time. DNGM introduces non-homogeneous terms based on GM to better accommodate nonlinear changes in the data, but is again limited by the static framework. MGM (Dynamic Multivariable Grey Model) is an important turning point. It makes the model framework dynamic, allowing the model to integrate new data points in real-time and dynamically adjust internal parameters to track new trends or fluctuations in the data effectively. This dynamic nature greatly improves the adaptability and prediction accuracy of the model when dealing with dynamically changing data. The dynamic gray model's primary strength lies in integrating and promptly responding to newly available data. When new information is or observed data are available, the dynamic gray model can adjust its internal parameters to reflect the current data state and potential trends. This dynamic adjustment mechanism ensures that the model can maintain high prediction accuracy in the face of rapid changes in data.

Page 27, lines 11-18: (The superiority of the new model)

DNMGM model, as the core innovation of this study, integrates the nonlinear processing capability of DNGM and the dynamic characteristics of MGM to build an efficient prediction model that can not only capture the nonlinear characteristics of data but also respond to data changes in real-time. This combination significantly improves DNMGM's accuracy and computational efficiency compared with other models, especially when dealing with complex and variable construction death data, and its superiority has been fully verified.

Page 27-28, lines 18-28 & 1-12: (A comparative description of the accuracy and computational efficiency of the new models)

In terms of accuracy, the DNMGM (1,1) model can effectively adapt to the characteristics of large fluctuations, seasonality, and strong trend of data in the construction field through the nonlinear transformation and dynamic adjustment mechanism so that the prediction accuracy is often better than some simple linear regression models or machine learning algorithms that do not fully consider the dynamic characteristics of data—for example, basic ANN (Artificial Neural Network) performance before complex tuning. However, compared to more advanced machine learning algorithms such as RF (Random Forest), GBT (Gradient Lift Tree), or deep learning models such as LSTM (Long Short Term Memory Network), the accuracy of DNMGM (1,1) can vary depending on the characteristics of the data. When the data amount is large enough, and the feature relationship is complex, these algorithms may show higher prediction accuracy through more complex nonlinear mapping and stronger learning ability. Regarding computing efficiency, the DNMGM (1,1) model usually has a faster computing speed and lower resource consumption due to its concise model structure and fewer parameter Settings, which is suitable for application scenarios with high real-time requirements. In contrast, although machine learning algorithms can provide higher prediction accuracy, their computational complexity is often higher, requiring more computational resources and time for model training and prediction, which may be a limiting factor in some situations where real-time performance is strictly required.

Page 28, lines 13-21: (Summary and significance)

In general, GM and DNGM models demonstrate the advantages of grey system theory in dealing with uncertainty and small sample data, but the static framework limits them. The MGM model overcomes this limitation by introducing dynamics. DNMGM model further combines the advantages of nonlinear and dynamic, providing a new perspective and tool for prediction research in building construction safety. The differences and similarities of these models reflect the development of grey system theory and provide valuable references and enlightenment for future research.

**Response 6-(4):** Thank you very much for your valuable suggestions, and based on your comments, I have revised the relevant content in order to more clearly elaborate the dynamic adaptability and generalisability of the DNMGM(1,1) model, so as to enhance the comprehension and depth of the article.

**Reviewer #2:**

**Comment 1:**

*1.The use of the dynamic nonhomogeneous gray model (DNMGM(1,1)) represents a significant advancement in predictive modeling for urban resilience. The ability of this model to integrate new data in real time enhances its applicability in rapidly changing urban environments.*

**Response 1：**

I am deeply grateful for your recognition of the DNMGM(1,1) model as a pivotal advancement in predictive modeling for urban resilience. Your acknowledgment of its real-time data integration capability underscores its adaptability to dynamic urban environments, which aligns perfectly with our aspirations for enhancing predictive accuracy and applicability.

**Comment 2:**

*2.The study effectively validates the DNMGM(1,1) model using real-world data on construction and traffic accident fatalities. The reported average relative errors demonstrate the model's superior accuracy compared to traditional gray models, reinforcing its potential for practical implementation.*

**Response 2：**

Your commendation of the validation process using real-world data on construction and traffic fatalities is highly appreciated. The reported average relative errors indeed serve as a testament to the DNMGM(1,1) model's superior accuracy over traditional gray models, reinforcing its practical significance and potential for widespread adoption.

**Comment 3:**

**Comment 3-(1):** *What specific parameters were used in the DNMGM(1,1) model, and how were they determined?*

**Comment 3-(2):** *Could the choice of these parameters affect the model's predictive accuracy?*

**Response 3：**

**Response 3-(1):** Thank you for your advice. In response to your questions about the specific parameters in the DNMGM(1,1) model and how they are determined, our responses are as follows:

In the DNMGM(1,1) model, the parameters used are not pre-set fixed values, but are mathematically calculated based on historical data. These parameters mainly include MAPE, which describes the uncertainty and randomness in the system. The process of determining these parameters involves additive generation (AGO) processing of the original data series, as well as solving the model parameters through optimization methods such as least squares to ensure that the model can best fit historical data and predict future trends.

Page 17, lines 4-7:

In this study, MAPE (Mean Absolute Percentage Error) was selected as the core index to quantify and evaluate the applicability of each model in predicting the number of deaths from construction accidents in China.

**Response 3-(2):** In addition, your consideration is very reasonable, the choice of parameters is crucial to the predictive performance of the model. The accurate calculated parameters can reflect the intrinsic law of data series more truly, to improve the model's prediction accuracy. On the contrary, if the parameter calculation is not accurate or improperly selected, it may cause the model prediction results to deviate from the actual, reducing the prediction's accuracy. Therefore, in the application of the DNMGM(1,1) model, we attach great importance to the accurate calculation and reasonable selection of parameters to ensure that the model can provide reliable prediction results.

Page 17, lines 7-10:

As an assessment indicator, MAPE helps quantify the model prediction accuracy, visually reflects the degree of prediction deviation from the true value through a percentage, and has a guiding role in improving the model prediction performance.

To sum up, parameters in the DNMGM(1,1) model are calculated through data, and their selection has an important impact on the model's prediction accuracy. We are committed to using scientific methods to determine model parameters to support accurate urban planning and disaster management decision-making.

**Comment 4:**

*4. What were the primary sources of the data used for model validation? How was data quality ensured, and were there any significant challenges in obtaining reliable data for this study?*

**Response 4：**

Thank you very much for your advice. In order to enhance the credibility of the data and ensure its quality, we explained the source of the data before the data analysis and processing. There are no major challenges in collecting the data， because the data is officially published and easily available.

Page 15, lines 6-15:

The data used in this paper are from the official statistical yearbook of the Ministry of Housing and Urban-Rural Development of China. The statistical yearbook is regularly compiled and released by authoritative institutions, aiming at comprehensively and systematically reflecting the development status, changing trends and existing problems in the field of housing and urban and rural construction in China. Our data is carefully screened and collated to ensure its accuracy, authority and timeliness. These data not only provide a solid foundation for the research of this paper, but also further enhance the credibility and persuasiveness of the research conclusions.

**Comment 5:**

*5. The research underscores the versatility of the DNMGM(1,1) model by demonstrating its efficacy in different domains, such as construction and traffic safety. This highlights the model's robustness and potential for widespread use in various aspects of urban planning and risk management.*

**Response 5：**

Your observation on the DNMGM(1,1) model's versatility across diverse domains, including construction and traffic safety, underscores its robustness and generalizability. This multifaceted applicability is indeed a testament to the model's strength and paves the way for its integration into various aspects of urban planning and risk management strategies.

**Comment 6:**

*6.How does the DNMGM(1,1) model compare with other advanced predictive models, such as machine learning algorithms, in terms of accuracy and computational efficiency? Are there scenarios where other models might be more suitable?*

**Response 6：**

Thank you very much for your advice. In response to your suggestion, we have included a description of accuracy and computational efficiency in our summary of the DNMGM(1,1) model. Here is what we added:

Page 27-28, lines 18-28 & 1-12:

In terms of accuracy, the DNMGM (1,1) model can effectively adapt to the characteristics of large fluctuations, seasonality, and strong trend of data in the construction field through the nonlinear transformation and dynamic adjustment mechanism so that the prediction accuracy is often better than some simple linear regression models or machine learning algorithms that do not fully consider the dynamic characteristics of data—for example, the performance of basic ANN (Artificial Neural Network) before complex tuning. However, compared to more advanced machine learning algorithms such as RF (Random Forest), GBT (Gradient Lift Tree), or deep learning models such as LSTM (Long Short Term Memory Network), the accuracy of DNMGM (1,1) can vary depending on the characteristics of the data. When the data amount is large enough, and the feature relationship is complex, these algorithms may show higher prediction accuracy through more complex nonlinear mapping and stronger learning ability. Regarding computing efficiency, the DNMGM (1,1) model usually has a faster computing speed and lower resource consumption due to its concise model structure and fewer parameter Settings, which is suitable for application scenarios with high real-time requirements. In contrast, although machine learning algorithms can provide higher prediction accuracy, their computational complexity is often higher, requiring more computational resources and time for model training and prediction, which may be a limiting factor in some situations where real-time performance is strictly required.

**Comment 7:**

*7.Given the model's sensitivity to abnormal data fluctuations, what specific strategies or combination methods are being considered to enhance its robustness? How might these strategies be implemented in future research?*

**Response 7：**

Thank you very much for your pertinent advice. We have revised and explained the limitations in the conclusion:

Page 30, lines 9-16:

However, the predictive effect of the DNMGM(1,1) model may be impacted by abnormal data fluctuations resulting from natural disasters, major incidents, or policy changes. To enhance the model's robustness and adaptability, specific strategies should be considered including, but not limited to, incorporating outlier detection and handling mechanisms and exploring hybrid approaches by combining the DNMGM(1,1) model with other prediction methods or algorithms. Future research should delve deeper into the design and implementation of such combination methods, aiming to make the DNMGM(1,1) model more resilient and applicable across various domains.

**Comment 8:**

*8. It is suggested to add articles entitled "Baxhuku et al. New Law Enforcement Impact on the Prevention of Road Accidents in Kosovo," "Balal et al. Forecasting Solar Power Generation Utilizing Machine Learning Models in Lubbock" and "Al-Abayechi & Al-Khafaji. Forecasting the Impact of the Environmental and Energy Factor to Improve Urban Sustainability by Using (SEM)" to the literature review.*

**Response 8：**

Thank you very much for your advice. After carefully reading the references you provided, we believe these articles will greatly enrich our literature review section. We have added these articles to our literature review. The details are as follows:

Page 5, lines 14-16:

In addition, through other angles, such as strengthening safety law enforcement, safety supervision, etc., can also effectively prevent the occurrence of accidents [5].

Page 7-8, lines 24-28 & 1-3:

Researchers are constantly exploring new prediction methods, such as SEM (structural equation model) [46], which combines the characteristics of factor analysis and path analysis, and RF algorithm (Random Forest) [47], which can simultaneously process multiple observed variables and potential variables, and construct multiple decision trees and summarize their prediction results to improve prediction accuracy and stability. However, they usually have the disadvantage of high computational cost.

**Comment 9:**

*9. The conclusion suggests future research should focus on combining the DNMGM(1,1) model with other predictive methods to address its limitations in handling abnormal data fluctuations. This proactive approach is commendable and necessary for enhancing the model's reliability and applicability.*

**Response 9：**

Thank you for your recognition. This proactive approach aligns with our commitment to continually improve the model's reliability and applicability, ensuring it remains a valuable tool in the face of complex and ever-evolving urban challenges.

**Comment 10:**

*10. The study presents its findings in a clear and structured manner, making it easy to follow the progression from model development to validation and application. This clarity is essential for understanding the model's capabilities and limitations.*

**Response 10：**

I am heartened by your praise for the clarity and structured presentation of our findings. We believe that a well-organized narrative, from model development to validation and application, is crucial for fostering a comprehensive understanding of the DNMGM(1,1) model's capabilities and limitations. Your appreciation of this aspect underscores the importance of clear communication in advancing scientific knowledge and fostering practical applications.

**Comment 11:**

**Comment 11-(1):** *What are the practical implications of implementing the DNMGM(1,1) model in urban planning and disaster management?*

**Comment 11-(2):** *Are there any case studies or pilot projects that demonstrate its real-world applicability and benefits?*

**Response 11：**

**Response 11-(1):** Thank you very much for your advice. We made a supplementary explanation of the meaning at the end of 4.1.

Page 25-26, lines 11-17 & 1-3:

A key characteristic of resilient cities is their ability to adapt to change dynamically. The DNMGM(1,1) model can update historical information to reflect new data, which means that forecasts are not static but can be adjusted based on the most recent data. In urban planning and disaster management, it is not limited to a single dimension of construction safety, but as an important tool for urban resilience construction, it can assist decision-making across fields and dimensions. By updating historical information in real time to reflect the latest data changes, DNMGM(1,1) model gives cities the ability to dynamically adapt to challenges, enabling resource allocation and risk response strategies to be flexibly adjusted according to the actual situation, which is of immeasurable value for improving cities' ability to cope with emergencies and ensuring residents' safety and well-being.

**Response 11-(2):** For the second question, although the direct case study is a strong evidence to prove the effectiveness of the model, considering the advantages of the existing literature and the theory of the model, the reasoning analysis combined with the actual situation can also support its potential and benefits in practical application. Therefore, this paper does not directly cite case studies, but elaborates the practical application value of DNMGM(1,1) model through theoretical analysis and existing research results.
